# Serum Resistin and Kidney Function: A Family-Based Study in Non-Diabetic, Untreated Individuals

**DOI:** 10.1371/journal.pone.0038414

**Published:** 2012-06-12

**Authors:** Claudia Menzaghi, Lucia Salvemini, Grazia Fini, Ryan Thompson, Davide Mangiacotti, Rosa Di Paola, Eleonora Morini, Maddalena Giorelli, Concetta De Bonis, Salvatore De Cosmo, Alessandro Doria, Vincenzo Trischitta

**Affiliations:** 1 Research Unit of Diabetes and Endocrine Disease, IRCCS Casa Sollievo della Sofferenza San Giovanni Rotondo, Italy; 2 Research Division, Joslin Diabetes Center, Boston, Massachusetts, United States of America; 3 Unit of Endocrinology, IRCCS Casa Sollievo della Sofferenza, San Giovanni Rotondo, Italy; 4 Department of Medicine, Harvard Medical School, Boston, Massachusetts, United States of America; 5 Mendel Laboratory, IRCSS Casa Sollievo della Sofferenza, Rome, Italy; 6 Department of Experimental Medicine, Sapienza University of Rome, Rome, Italy; Universita Magna-Graecia di Catanzaro, Italy

## Abstract

**Background:**

High serum resistin levels have been associated with kidney dysfunction. Most of these studies have been carried out in individuals with severe kidney impairment, diabetes, cardiovascular disease and related treatments. Thus, the observed association might have been influenced by these confounders. Our aim was to study the relationship between serum resistin, urinary albumin/creatinine ratio (ACR) and glomerular filtration rate (GFR) in a family-based sample, the Gargano Family Study (GFS) of 635 non diabetic, untreated Whites.

**Methods:**

A linear mixed effects model and bivariate analyses were used to evaluate the phenotypic and genetic relations between serum resistin and both ACR and eGFR. All analyses were adjusted for sex, age, age squared, BMI, systolic blood pressure, smoking habits and physical exercise.

**Results:**

After adjustments, resistin levels were slightly positively associated with ACR (β±SE = 0.049±0.023, p = 0.035) and inversely related to eGFR (β±SE = −1.43±0.61, p = 0.018) levels. These associations remained significant when either eGFR or ACR were, reciprocally, added as covariates. A genetic correlation (ρg = −0.31±0.12; adjusted p = 0.013) was observed between resistin and eGFR (but not ACR) levels.

**Conclusion:**

Serum resistin levels are independently associated with ACR and eGFR in untreated non-diabetic individuals. Serum resistin and eGFR share also some common genetic background. Our data strongly suggest that resistin plays a role in modulating kidney function.

## Introduction

Kidney dysfunction is a worldwide public health concern and a major risk factor for end-stage renal disease, cardiovascular events and premature death [1]. Identifying and treating risk factors for this abnormality may be a valuable approach to prevent such devastating clinical outcomes [1]. Insulin resistance, inflammation and endothelial dysfunction have been recognized, among other factors, as prime movers of kidney dysfunction [2], [3], [4]. Recently, new molecules, secreted by adipose tissue and known as adipokines, have been linked to all the above mentioned conditions [5]. Among these is resistin, a 12.5 kDa cysteine-rich protein which is also abundantly secreted by macrophages [6]. High serum resistin levels have been associated with kidney dysfunction, including low glomerular filtration rate (GFR) and increased albuminuria, in several studies [7], [8], [9], [10], [11], [12], [13]. However, most of these data were obtained in cohorts with severe kidney impairment and cardiovascular disease and with a high proportion of diabetic patients [7], [8], [9], [10], [11], [12]. Of note, the only study in the general population was carried out in Japanese individuals among whom, however, hypertension was highly prevalent (i.e. almost 50%) [13]. Thus, the observed associations might have been influenced by any of these abnormalities as well as their related treatments (i.e. thiazolinediones, statins and anti-hypertensive drugs), which are known to affect circulating resistin levels [14], [15], [16].

The aim of the present study was to investigate the relationship between circulating resistin levels and renal function, as assessed by urinary albumin/creatinine ratio (ACR) and GFR in the absence of the above mentioned confounders. To pursue this aim a family-based sample, the Gargano Family Study (GFS) of 635 non-diabetic Whites individuals, not treated with medications known to interfere with glucose homeostasis, lipid profile, blood pressure and/or to modulate resistin and ACR levels were studied. We also addressed the issue of whether or not circulating resistin shares a common genetic background with either trait and, if so, if this was explained at least in part by two SNPs in the resistin gene *RETN* (i.e. rs1862513 and rs3745367) that have been previously associated with resistin levels [17], [18], [19].

## Materials and Methods

### Subjects

The GFS comprises a total of 635 non-diabetic White individuals, from 218 families recruited in the Gargano area (an homogeneous geographical area in Center-East Italy) examined as previously described [18], [20], [21]. Briefly, subjects were examined between 08:00 and 09:00 h after an overnight fast. Height, weight, waist and hip circumferences, and blood pressure were measured in duplicate, and a blood sample was drawn for biochemical measurements and DNA extraction.

All study subjects were not treated with medications known to interfere with glucose homeostasis, lipid profile, blood pressure and known to modulate resistin and ACR levels.

### Ethics

The study and the informed consent procedures were approved by the local Institutional Ethic Committee IRCCS (Istituto di Ricovero e Cura a Carattere Scientifico) “Casa Sollievo della Sofferenza”. All participants gave written consent.

### Measurements

Plasma glucose was measured by the glucose oxidase method on a Beckman Glucose Analyzer 2 (Beckman Coulter, Inc., Fullerton, CA), serum insulin was measured by microparticle enzyme immunoassay (Abbott IMx Insulin Assay, Abbott Laboratories, Abbott Park, IL), and lipid profile (total serum cholesterol, HDL cholesterol and serum triglycerides) were measured by enzymatic method, Cobas, Roche Diagnostic, Welwin Garden City, Herts, UK.

Serum resistin concentrations were measured by a commercial ELISA kit (Bio Vendor, Brno Czech Republic). Inter- and intra-assay coefficients of variation were 3.2–4% and 6.3–7.2%, respectively [18].

Urinary albumin and creatinine concentrations were determined the same morning of the clinical examination on an early morning first void sterile urine sample by the nephelometric method (Behring Nephelometer Analyzer) and the Jaffè reaction-rate method (Hitachi 737 Autoanalyzer), respectively. Elevated urinary albumin excretion was diagnosed if the ACR was ≥2.5 mg/mmol in men and ≥3.5 mg/mmol in women.

GFR (eGFR) was estimated by CKD-EPI creatinine formula [22].

The insulin resistance index homeostasis model assessment (HOMA_IR_) was calculated as fasting serum insulin (pmol/liter) x fasting plasma glucose (mmol/liter)/135 [18], [20], [21].

### Genotyping

SNPs rs1862513 and rs3745367 in the *RETN* gene, selected because of their previous association with resistin circulating levels [17], [18], [19] were genotyped as previously described [18]. In addition, for the *RETN* gene (i.e. 1,369 bp), rs3745367 is the only tag SNP described for CEU population (phase 2+3 HapMap database -www.hapmap.org- February 2009). Call rate and concordance rate were >98% and >99% respectively. Out of 635 study individuals, genotypes were available for 628 study subjects for rs1862513 and for 627 study subjects for rs3745367. Allele and genotype frequencies were as follows. For rs1862513, C: 68.1% and G: 31.9%; CC: 47.6%, CG: 41% and CC: 11.4%, respectively. For rs3745367, G: 68.6% and A: 31.4%; GG: 48.2%, GA: 40.8% and AA: 11%, respectively. Both SNPs were in Hardy–Weinberg Equilibrium (HWE) (p>0.05).

### Statistical Analysis

Data are summarized as means±SD and median (range). Because of resistin and ACR skewness, logarithmic transformation was performed before further analyses.

Since we aimed at unraveling possible overall genetic correlation between serum resistin and kidney function that cannot be obtained from unrelated individuals, we analyzed nuclear families which, in contrast, allow test such hypothesis.

To determine the contribution of genetic factors to serum resistin, the SOLAR software package (Version 4.1.7) was utilized [23]. SOLAR performs a variance components analysis of family data that decomposes the total variance of the phenotypes into components that are due to genetic effects (i.e. polygenic, additive genetic variance), measured covariates, and random environmental effects (i.e. unique, unshared environmental effects). [24] The relative contribution of genetic factors to serum resistin is then estimated by heritability (h^2^), defined as the ratio of the genetic variance component to the residual (after removal of covariates) phenotypic variance. Heritability estimates, so obtained, also include any environmental contributions to similarities in adjusted values between relatives [24].

To assess phenotypic correlations between resistin and ACR and GFR, we used a mixed effects model implemented in SOLAR [23] that includes fixed and random effects. In our variance components model, the fixed effects are the covariates (i.e. sex, age, age squared, BMI, systolic blood pressure smoking habits and physical exercise), to account for known variation by these factors. The random effects are defined by partitioning the covariance between relative pairs into additive genetic and error covariance. The covariance structure was determined by the degree of relatedness between each relative. This method accounts for the dependence of the family data and provides more stringent p values. Results were reported as linear model coefficients along with their standard errors and p-values (β±SE, p). P values<0.05 after correction for the two comparison we made for each model, according to Hochberg’s method [25], were considered to be significant.

In a multivariate model, bivariate analyses were conducted to partition the phenotypic correlation between two traits (ρp) into genetic (ρg), due to shared additive genetic effects(i.e. pleiotropy) and environmental (ρe, due to shared random environmental effects) correlations [24]. Thus, the phenotypic correlation is influenced by both the genetic and environmental correlations where each is weighted by the proportion of variation due to genes (genetic) and environmental factors (non-genetic), respectively. This approach has been implemented in SOLAR [26]. The phenotypic correlations after the familial correlation was accounted for are derived from the equation ρp = ρg√*h_1_^2^*√*h_2_^2^+*ρe √ (1−*h_1_^2^)* √ (1−*h_2_^2^)* where *h*_1_^2^ and *h*_2_^2^ correspond to the heritability of traits 1 and 2, respectively. Evidence of pleiotropy (i.e. a common set of genes influencing more than one trait) is indicated by a genetic correlation significantly different from zero. Shared environmental factors are indicated by an environmental correlation significantly different from zero.

## Results

Clinical characteristics of study participants from the GFS are shown in Table 1.

**Table 1:** Clinical characteristics of study participants of the GFS (635 non-diabetic individuals from 218 families).

	Mean±SD	Median (Range)
M/F	246/389	
Age (yrs)	40.1±14.4	40.0 (16–82)
BMI (Kg/m^2^)	26.3±4.7	25.5 (17.1–48.2)
Waist circumference (cm)	84.7±12.5	84.0 (50–126.0)
Obese (%)	18.6	
Overweight (%)	10.2	
SBP (mmHg)	116.9±14.5	115.0 (80–180)
DBP (mmHg)	77.1±9.0	80.0 (50–112)
Hypertensive (%)	9.8	
FBG (mmol/L)	5.0±0.57	4.89 (3.20–6.99)
Insulin (pmol/L)	55.6±31.9	49.3 (12.5–333.4)
HOMA_IR_	1.8±1.1	1.54 (0.36–10.0)
Triacylglycerol (mg/dL)	100.3±66.1	81.0 (28.0–520.0)
HDL cholesterol (mg/dL)	52.8±13.3	52.0 (21–119.0)
eGFR	87.97±13.4	87 (60–184)
ACR (mg/mmol)	0.9±1.16	0.53 (0.06–12.4)
Fibrinogen (mg/dL)	292.8±61.4	286.0 (149–591)
Resistin (ng/mL)	5.9±3.0	5.3 (1.2–26.8)

Data are expressed as Mean ± SD or %.

BMI: Body Mass Index; SBP: Systolic Blood Pressure; DBP: Diastolic Blood Pressure; FBG: Fasting Blood Glucose; HOMA_IR_: homeostasis model assessment of insulin-resistance; HDL-Cholesterol: high-density lipoprotein cholesterol; eGFR: estimated Glomerular Filtration Rate by CKD-EPI formula; ACR: Albumin Creatinine Ratio.

Obese: BMI ≥30.

Overweight: BMI ≥25≤29.9.

Hypertensive: (i.e. systolic blood pressure ≥140 mmHg and/or diastolic blood pressure ≥90 mmHg).

This study comprises 140 nuclear families, 75 sibships and 20 extended sibships (ranging 3–5 individuals).

As previously reported [18] in this set, after adjusting for sex, age, age squared, smoking habits and physical exercise, serum resistin was highly heritable, (*h* ^2^ = 73.7±0.08 *P = *6.05×10^−18^).

After adjusting for sex, age, age squared, BMI, systolic blood pressure, smoking habits and physical exercise, serum resistin levels were slightly positively associated with ACR (β±SE = 0.049±0.023, p = 0.035) and inversely related to eGFR levels (β±SE = −1.43±0.61, p = 0.018) (Table 2).

**Table 2:** Association of serum resistin levels and renal functions in the GFS.

	ACR (mg/mmol)			eGFR (mL/min)		
	β±SE	p	p*	β±SE	p	p*
Model 1	0.049±0.023	0.035	0.035	−1.43±0.61	0.018	0.035
Model 2	0.048±0.023	0.048	0.048	−1.24±0.39	0.0067	0.0134
Model 3	0.047±0.023	0.047	0.047	−1.26±0.36	0.016	0.0320
Model 4	0.046±0.023	0.049	0.049	−1.29±0.45	0.013	0.026

The linear β coefficients represent the change in ACR and GFR levels for 1 unit increase of serum resistin levels (ng/mL).

ACR: Albumin Creatinine Ratio; eGFR: estimated Glomerular Filtration Rate by CKD-EPI formula.

Model 1: analyses are adjusted for sex, age, age squared, BMI, smoking habit, physical exercise and systolic blood pressure.

Model 2: model 1 plus adjustment for eGFR or ACR when testing the association of resistin with ACR and GFR, respectively.

Model 3: model 1 plus adjustment for HOMA_IR._

Model 4: model 1 plus adjustment for plasma fibrinogen.

* P value obtained after Hochberg correction.

The associations between resistin and ACR (β±SE = 0.048±0.023, p = 0.048) as well as that between resistin and eGFR (β±SE = −1.24±0.39, p = 0.0067) remained statistically significant when eGFR and ACR were reciprocally used as additional covariates (Table 2). Similarly, results did not change much when the insulin resistance index, HOMA_IR_, or the inflammatory marker fibrinogen were also added to the model (Table 2). All the association remained statistically significant after Hochberg correction (Table 2).

A significant inverse genetic correlation was observed between resistin and eGFR (ρg = −0.31±0.12; p = 0.013), but not between resistin and ACR (data not shown).

Among the two *RETN* SNPs studied only SNP rs3745367, previously associated with resistin levels in the same setting [18] explains some phenotypic variation (1.5%) for serum resistin. By contrast SNP rs1862513 in our sample is not associated with resistin (p = 0.73). Neither SNP rs3745367 nor SNP rs1862513 were associated with ACR (p = 0.39 and p = 0.56, respectively) or eGFR (p = 0.41 and p = 0.53, respectively).

No significant environmental correlations were found between resistin and kidney function traits (Table S1).

Sixty-two out of 635 individuals (9.8% of the whole sample) were diagnosed as having hypertension (i.e. systolic blood pressure ≥140 mmHg and/or diastolic blood pressure ≥90 mmHg). Exclusion of these individuals did not affect any of the results described above (data not shown).

## Discussion

The main finding of our study is that serum resistin is inversely and independently associated with eGFR and shares with it a common genetic (but not environmental) background.

Importantly, of all the variables we had the opportunity to test in our study, resistin is by far the most important predictor of eGFR, accounting for 7% of its variance. Taken together, our data suggest that resistin is a strong modulator of kidney function.

We also observed a weaker association between resistin and ACR. However, the level of statistical significance of this association is marginal making the relevance of this finding uncertain.

Unfortunately we were unable to asses if the established notion of a common genetic background between serum resistin and GFR could be explained by some variation at the *RETN* locus. In fact, of the two SNPs we investigated, the only one which was associated with serum resistin (i.e. rs3745367), was not associated with eGFR.

It must be emphasized that the sample that we analyzed consists only of non-diabetic, untreated, relatively young individuals (mean age = 40 years) with normal kidney function and no clinical reports of cardiovascular disease. Thus, our results are not influenced by the possible confounders that may have heavily affected previous studies addressing the role of resistin on kidney function [7], [8], [9], [10], [11], [12], [13].

In addition, we choose a family-based study for our specific purpose, because it is of note that, in general, such studies have more statistical power and minimize the risk for population stratification, as compared to studies carried out in unrelated individuals [27].

Resistin has been linked both with insulin resistance and inflammation [6]; however, our data showing that the association between serum resistin and eGFR is not affected by either HOMA_IR_ or plasma fibrinogen, suggest that it is not mediated by systemic insulin resistance or chronic low-grade inflammation. It has been recently reported that resistin may play an important role in potentiating endothelial dysfunction through oxidative stress [28]. One can then speculate that resistin may have a direct deleterious effect on the glomeruli, as shown for other adipokines [29]. It is also possible that other, as yet unidentified systemic pathways underlie the biology of the association between resistin and kidney function.

This study has some limitations. We did not investigate comprehensively the impact of *RETN* locus genetic variability in modulating the common genetic background shared by resistin and eGFR, limiting our genotyping only to two SNPs which have been previously associated with serum resistin levels in our [18], [19] and other populations [17]. So, we cannot exclude that other SNPs outside the *RETN* gene play also a role in modulating the common genetic background between resistin and eGFR. In addition, whether our data can be generalized to other populations with different environmental and/or genetic background is not known and deserves further investigation [30].

In conclusion, our study shows a strong and independent correlation between resistin levels and GFR. The two variables share also some common genetic background. Dissection of the exact mechanisms underlying this relationship may help develop tailored interventions aimed at preventing kidney function loss in high risk individuals.

## Supporting Information

Table S1 Genetic, environmental and phenotypic correlations between serum resistin levels and kidney functions in the GFS.
(DOC)
