# Supplementary material for: Serum Resistin and Kidney Function: A Family-Based Study in Non-Diabetic, Untreated Individuals
Source: PLoS One. 2012 Jun 12;7(6):e38414. doi: 10.1371/journal.pone.0038414 (PMC3373540; doi:10.1371/journal.pone.0038414)
Supplement: Table S1 — Genetic, environmental and phenotypic correlations between serum resistin levels and kidney functions in the GFS. (DOC) [file pone.0038414.s001.doc]

**Table S1. Genetic, environmental and phenotypic correlations between serum resistin levels and kidney functions in the GFS**

|  | Serum resistin | | | | |
| --- | --- | --- | --- | --- | --- |
|  | ρg±SE | p | ρe±SE | p | ρp |
| ACR | -0.18±0.19 | 0.93 | -0.27±0.12 | 0.71 | 0.026 |
| eGFR | -0.31±0.12 | 0.013 | -0.29±0.17 | 0.062 | -0.06 |

GFS = Gargano Family Study.

ρg = Genetic correlation.

ρe = Environmental correlation.

ρp=phenotypic correlation, as calculated from ρg and ρe according to the equation: ρp = ρg√*h_1_^2^*√*h_2_^2^ +* ρe √ (1-*h_1_^2^)* √ (1-*h_2_^2^)* where *h*_1_^2^ and *h*_2_^2^ correspond to the heritability of traits 1 and 2, respectively.

All analyses are adjusted for age, age^2^, gender, smoking habits and physical exercise.
